# Supplementary material for: Pharmacist-led medication reviews: A scoping review of systematic reviews
Source: PLoS One. 2024 Sep 6;19(9):e0309729. doi: 10.1371/journal.pone.0309729 (PMC11379247; doi:10.1371/journal.pone.0309729)
Supplement: S1 File — (DOCX) [file pone.0309729.s001.docx]

# Supplementary file 1

**Research strategy for Scoping Review**

The OVID platform was used to search the Embase and MEDLINE databases. This meant that only one search string was necessary.

The following search terms were used:

pharmac* AND [“medicine review” OR “medication review” OR “medicines review”] AND “systematic review”. The search was restricted to abstracts; these, along with their titles, were reviewed, and full text retrieved of those that met the inclusion criteria. Through forward and backward reference searching, other papers of interest were identified. In addition, the Cochrane database of systematic reviews was searched for relevant systematic reviews published after December 2015 (‘medication review’ and ‘pharmacist’).

A time filter was applied to the results: January 2016 to January 2023 (the time of the search).
