# Supplementary material for: Pharmacist-led medication reviews: A scoping review of systematic reviews
Source: PLoS One. 2024 Sep 6;19(9):e0309729. doi: 10.1371/journal.pone.0309729 (PMC11379247; doi:10.1371/journal.pone.0309729)
Supplement: S1 Table — (DOCX) [file pone.0309729.s002.docx]

| **S2 Table 1 Description of systematic reviews and AMSTAR 2 ratings** | | | | | | | | | |
| --- | --- | --- | --- | --- | --- | --- | --- | --- | --- |
|  | | | | | | | | | |
| **Author, year** | **Research Question** | **Study design & number** | | **Population & setting** | **Countries of origin** | **Meta-analysis** | **AMSTAR 2** | **Authors’ outcomes of interest** | **Main results** |
| Post 2015 systematic reviews | | | | | | | | | |
| Alldred 2016 [16] | What is the effect of interventions to optimise overall prescribing for older people living in care homes? | RCT n=12 | | People >65 years; Institutionalised care facilities | Australia, Finland, Israel, Netherlands, New Zealand, Spain, Sweden, UK, USA, and Canada | No | High | Adverse drug events | No effect  2/2 studies report evidence of no effect |
|  |  |  |  |  |  |  |  | Hospital admissions | Uncertain effect  4/8 studies report reduction (these studies had flaws in their study design)  4/8 studies report evidence of no effect |
|  |  |  |  |  |  |  |  | Mortality | No effect  6/6 studies reported evidence of no effect |
|  |  |  |  |  |  |  |  | Quality of life (QoL) | Uncertain effect  1/ 2 studies reported no evidence for effect  1/ 2 studies reported a slower decline in QoL (this study had flawed statistical analysis) |
|  |  |  |  |  |  |  |  | Medication-related problems (MRPs) | Evidence of effect: resolution of MRPs  7/7 studies reported an increase in identified MRPs, but these were diversly classified leading to lack of consistency and reliability of results |
|  |  |  |  |  |  |  |  | Medication appropriateness | Evidence of effect: medication appropriateness  5/5 studies reported a reduction in potentially inappropriate medicines |
|  |  |  |  |  |  |  |  | Medicine costs. | Uncertain effect: medicine costs  3/5 studies reported reduction in costs  2/5 studies reported no difference |
| Bulow 2023 [21] | Does delivery of a medication review by a physician, pharmacist or other healthcare professional leads to greater improvement in health outcomes of hospitalised adult patients? | RCT n=25 | | Adults;  hospital | Sweden, Denmark, Belgium, USA, Ireland, Northern Ireland, Switzerland, Brazil, Canada, Netherlands, Norway, Germany, Scotland, Spain, Italy, Iceland, South Korea | Yes | High | Mortality | No effect: mortality  18 studies, 10108 participants  **Risk ratio 0.96 [ 0.87, 1.05]** |
|  |  |  |  |  |  |  |  | Hospital readmission | Evidence of effect: readmission reduction  17 studies, 9561 participants  **Risk ratio 0.93 [ 0.89, 0.98]** favouring MR |
|  |  |  |  |  |  |  |  | Hospital emergency department contacts | Evidence of effect: reduction in contacts  8 studies 3527 participants  **Risk ratio 0.84 [ 0.68, 1.03]** favouring MR |
|  |  |  |  |  |  |  |  | Health-related Quality of life | Uncertain evidence: change in QoL  4 studies 392 participants  **Mean reduction 0.10 [ -0.10, 0.30]** favouring MR |
| Al- babtain 2022 [20] | Impact of community-pharmacist-led medication review programmes on patient outcomes: A systematic review and meta-analysis of randomised controlled studies | RCT n=40 | | Adult patients; community pharmacy | USA, Canada, Netherlands, Australia, UK, Spain, Germany, Iran, Jordan, Croatia, Iraq, Malta, Portugal, Italy, Denmark | Yes | Moderate | Clinical outcomes measure | No effect: Mortality  3 studies  2/3 studies reported a slight decrease  1/3 study reported a slight increase |
|  |  |  |  |  |  |  |  |  | Evidence of effect: Blood pressure reduction  MA of 9 studies 1642 participants  Pooled results Systolic BP **-6.56 (95% CI -10.05, -3.08) MD**  Pooled results Diastolic BP **-1.68 (95% CI -3.18, -0.18) MD** |
|  |  |  |  |  |  |  |  |  | Evidence of effect: Total cholesterol (reduction)  MA of 3 studies 379 participants  Pooled results **MD -0.18; [ -0.32, -0.05]; P=0.008; I^2^ =0%)** |
|  |  |  |  |  |  |  |  |  | Evidence of effect: HbA1c (reduction)  MA of 6 studies 1152 participants  Pooled results **(MD -0.61; 95% CI -0.96, 0.25,**  **P =0.0008** **I^2^ =0%)**). |
|  |  |  |  |  |  |  |  |  | Uncertain effect: Medicines Related Problems  6 studies  3/6 studies reported a reduction  1/6 study reported an increase  2/6 reported uncertain effect |
|  |  |  |  |  |  |  |  |  | Uncertain effect: Adverse Drug Events  4 studies  2/4 studies report a reduction  2/4 studies report an increase |
|  |  |  |  |  |  |  |  |  | Uncertain effect: Medication adherence  16 studies  15/16 studies reported an improvement  1/16 study reported a decrease |
|  |  |  |  |  |  |  |  |  | Uncertain effect: Quality of Life  16 studies  11/16 studies reported an improvement  2/16 studies reported an uncertain effect  3/16 studies reported a decline |
|  |  |  |  |  |  |  |  | Health service utilisation | Uncertain effect: Emergency department visits  5 studies  4/5 studies reported a reduction  1/5 study reported an increase |
|  |  |  |  |  |  |  |  |  | Uncertain effect: Hospital admissions/ re-admissions  9 studies  4/5 studies reported a reduction in hospital admission rate  1/5 study reported an increase in admission rate  2/4 studies reported a reduction in hospital admission days  1 /4 study reported an increase  1 /4 study reported an uncertain effect |
| Atey 2022 [34] | Does the delivery of interventions by pharmacists led to improved quality use of medicines for adult emergency department patients? | RCT n=3  Non-randomised studies n=28 | | Adults ≥ 18 years presenting to emergency department | USA, Belgium, Australia, UK, Colombia, Spain, Ethiopia, Taiwan, Egypt | Yes | Moderate | Medication errors | Evidence of effect: reduction in error rate  Pooled results of 12 studies 4959 participants  **Decrease of 0.33 per patient [95% CI -.42 to -0.23], p< 0.001** **I^2^ = 51%**  Evidence of effect: decrease in proportion of patients having at least one error  **MA of 10 studies 4742**  **RR=0.27, [CI 0.19 to 0.40], p< 0.001** **I^2^ = 89%** |
|  |  |  |  |  |  |  |  | Appropriateness of medications | Evidence of effect: increased appropriateness of prescribed medications  Pooled results of 7 studies 2613 participants  **RR=1.58 [CI 95% 1.21 to 2.06], p<0.001 I^2^ =95%** |
|  |  |  |  |  |  |  |  | Healthcare utilisation | No effect: length of hospital stay  Pooled results of 6 studies 1233 participants  Mean difference =0.28days [95% CI -0.88 to 1.45], p=0.018,  **I**^2^ =63.5%  Evidence of effect: reduced incidence of being re-admitted  Pooled estimated of 2 studies 291 participants  RR=0.62 [95% CI 0.38 to 0.99], p=0.05  Evidence of effect: reduction of re-presenting to emergency department  Pooled estimated of 2 studies 998 participants  **RR=0.70 [95% CI 0.52 to 0.94], p=0.02** |
| Fadaleh 2022 [22] | What is the effect of home medication review in community-dwelling older adults? | RCT n=18 | | Adults ≥65 years;  Community-dwelling | UK, Canada, Spain, Australia, USA, Denmark, Netherlands, Germany | Yes | Moderate | Healthcare utilisation | No effect: healthcare utilisation  MA of 9 studies of 3413 participants  **RR of 0.91 [95% CI 0.71, 1.15] I^2^ =85%** |
|  |  |  |  |  |  |  |  | Mortality | Uncertain effect: mortality  12/13 studies reported no effect  1/13 study reported a decrease in mortality |
|  |  |  |  |  |  |  |  | Medication outcomes | Uncertain effect: medication changes  5/7 studies report improvements in number of drugs prescribed  2/7 studies reported no difference  Uncertain effect: adherence  2/5 studies report [significant] improvement in adherence  3/5 studies report no change |
|  |  |  |  |  |  |  |  | Patient-orientated outcomes | Uncertain effect: Quality of Life  4/6 studies reported no effect  1/6 studies reported uncertain effect  1/6 studies reported improvement (statistically significant)  Uncertain effect: satisfaction  2/4 studies reported high patient satisfaction  2/4 studies reported no difference in satisfaction |
|  |  |  |  |  |  |  |  | Economic outcomes | Evidence of effect: cost savings  4/4 studies reported significant cost savings |
| Martinez-Mardones 2019 [19] | What is the impact of pharmacist-led MRs on CVD risk factors overall and in different ambulatory settings | RCT n=69 | | Patients with CV risk; ambulatory care, community pharmacy | Spain, Portugal, Chile, Brazil Australia, Canada, USA, Jordan, Egypt, Denmark, Cyprus, Hong Kong, China, Thailand | Yes | Low | CVD risk factors | Evidence of effect: Blood pressure reduction  MA of 31 studies 7031 participants.  **pooled OR of 2.73 (95% PI, 1.05–7.08)**  **I^2^ = 71%** |
|  |  |  |  |  |  |  |  |  | Evidence of effect : Type 2 diabetes Mellitus  MA of 25 studies 3452 participants  **OR for achieving control was 3.11 (95% PI, 1.48–6.52 I^2^=30%).** |
|  |  |  |  |  |  |  |  |  | Evidence of effect: Achieving cholesterol goals  MA of 11 studies 2012 participants  **OR of 1.91 (95% PI, 1.05– 3.46), I^2^=31%** |
| Ahumada-Canale 2019 [18] | Economic evaluations of pharmacist-led medication review in outpatients with hypertension, type 2 diabetes mellitus, and dyslipidaemia: a systematic review | RCT n=11 | | Adult patients;  community pharmacy, primary care centre, outpatient clinics | USA, Brazil, Canada, China, Nigeria, Taiwan, UK | No | Critically low | Health economics | Uncertain effect: Incremental costs per patient  9/11 studies reported in an increase in costs per patient  2/11 studies reported a deduction in costs per patient |
|  |  |  |  |  |  |  |  | CVD risk factors | Uncertain effect: Systolic blood Pressure  5/6 studies report a decrease  1/6 study report no effect |
|  |  |  |  |  |  |  |  |  | Uncertain effect: Diastolic blood pressure  5/6 studies report a decrease  1/6 study report no effect |
|  |  |  |  |  |  |  |  |  | Evidence of effect: BP goal  4/4 studies reported an increase in the number of patients achieving their treatment goal |
|  |  |  |  |  |  |  |  |  | Evidence of effect: Cardiovascular Risk  2/2 studies reported a reduction in cardiovascular risk |
|  |  |  |  |  |  |  |  |  | Evidence of effect: Life years gained  1/1 study reported life years gained for male and females |
|  |  |  |  |  |  |  |  |  | Evidence of effect: HbA1c  1/1 study reported improvement in control |
| Bou Malham 2021 [33] | Impact of pharmacist-led interventions on patient care in ambulatory care settings: A systematic review | RCT n=27  Observation studies n=4 | | Patients in outpatient settings; community pharmacy, home, hospital admission/ discharge, outpatient clinics, healthcare centres | UK, USA, Jordan, Europe | No | Critically low | Clinical | No effect: Potentially inappropriate prescriptions  1/1 study |
|  |  |  |  |  |  |  |  |  | Uncertain effect: Adverse Drug Events  6/7 studies reported a reduced incidence  1/7 study reported no effect |
|  |  |  |  |  |  |  |  |  | Evidence of effect: (reduction) Medicines related Problems  3/3 studies report increased resolution following intervention |
|  |  |  |  |  |  |  |  |  | Uncertain effect: Medication Appropriateness Index  2/3 studies report a reduction in the number of inappropriate medicines prescribed  1/3 study reported no effect |
|  |  |  |  |  |  |  |  | Economic | Uncertain effect: Cost  5/6 studies reported a reduction in healthcare costs  1/6 study reported no effect |
|  |  |  |  |  |  |  |  |  | No effect: Drug utilisation  1/1 study reported no effect |
|  |  |  |  |  |  |  |  |  | Uncertain effect: Secondary care service utilisation  5/10 studies reported in reduction in hospital attendance and admission  2/10 studies reported an increase in hospital attendance and admission  3/10 reported no effect on hospital attendance and admission |
|  |  |  |  |  |  |  |  | Patient-orientated | Uncertain effect: Adherence  6/7 studies reported an improvement in adherence  1/7 study reported no effect on adherence |
|  |  |  |  |  |  |  |  |  | Evidence of effect: Patient satisfaction  3/3 studies reported that patients were satisfied with the intervention |
|  |  |  |  |  |  |  |  |  | Uncertain effect: Quality of Life  3 /4 studies report no effect  1 /4 study reports an improvement |
|  |  |  |  |  |  |  |  |  | Evidence of effect: Symptom control  1/1 study reported an improvement in symptom control |
|  |  |  |  |  |  |  |  |  | Evidence of effect: Patient knowledge  1/1 study reported an increase in knowledge |
| Hikaka 2019 [23] | What are the components of medicines use review service models utilised in New Zealand? How effective are these interventions? | Retrospective case studies n=3  Prospective case studies n=2  Semi-structured interviews n=1 | | Adult patients;  Community pharmacy, home, outpatient clinic | New Zealand | No | Critically low | Medication-related Problems (MRPs) | Uncertain effect: number of MRPs  4/4 studies reported identification of MRPs  1/ 4 study reported a change in MRPs over time. This wasn’t reported upon in other studies |
|  |  |  |  |  |  |  |  | Adherence | Uncertain effect  1/3 study reported improved adherence over time  1/3 study reported reduced adherence  1/3 study did not clearly report results |
|  |  |  |  |  |  |  |  | Medicines knowledge | Evidence of effect  3/3 studies reported increased knowledge of medicines |
|  |  |  |  |  |  |  |  | Utilisation of secondary care services | No effect  1/1 study |
|  |  |  |  |  |  |  |  | Quality of life | Evidence of effect: Improvement in QoL Reported in 2 different tests  1/1 study |
|  |  |  |  |  |  |  |  | Patient satisfaction | Evidence of effect  3/3 studies reported that 78-93% of respondents were satisfied with their MR |
| Huiskes 2017 [17] | What is the evidence of medication reviews as performed in clinical practice? | RCTs n=31 | | All patient populations; all settings | UK, Australia, USA, Belgium, Netherlands, Sweden, Singapore, Denmark, Germany, Canada. | Yes | critically low | Clinical outcomes measure | No effect: clinical outcomes  However, 6/6 studies report decrease in the number of falls  MA of 4 studies 929 participants  **(RR 0.68 (0.52, 0.90); I^2^ = 41.0%, p=0.166).** |
|  |  |  |  |  |  |  |  |  | No effect: mortality  11 studies 2403 participants  **(RR 0.94 (CI, 0.76–1.17) I^2^ = 22.0%, P = 0.234)** |
|  |  |  |  |  |  |  |  |  | No effect: number of hospital admissions  11 studies 2041 participants  **(RR 0.94 (0.82, 1.08) I^2^ = 42.3%, P = 0.139)** |
|  |  |  |  |  |  |  |  |  | No effect: health status, physical and cognitive outcome measures.  3/3 studies reported evidence of no effect on physical functioning  2/3 studies reported no changes in clinical or health status  1/3 trial reported a smaller decrease in health following MR  2/2 studies reported no difference in cognitive functioning |
|  |  |  |  |  |  |  |  | Quality of life | Uncertain effect  3/8 studies using EQ-5D or SF-36 reported no difference in quality of life  5/8 studies using EQ-5D or SF-36 reported inconclusive results |
|  |  |  |  |  |  |  |  | Drug-related outcome measures | Uncertain effect: number of drug changes |
|  |  |  |  |  |  |  |  |  | Uncertain effect: number of medicines used |
|  |  |  |  |  |  |  |  |  | Uncertain evidence: impact on adherence, knowledge, and adverse effects |
| Jokanovic 2016 [32] | What are the processes and outcomes of CMR in community-settings in Australia? | RCT n=7,  non-RCT n=2, uncontrolled/ observational study n=34,  Qualitative study n=11,  survey research study n=9 | | All patient populations;  hospital, patient's home | Australia | No | critically low | Economical | Evidence for effect  2/2 studies reported MR was cost-effective |
|  |  |  |  |  |  |  |  |  | Uncertain effect: medication costs  4/6 studies reported reduced costs  2/6 studies reported no reductions |
|  |  |  |  |  |  |  |  | Clinical | Uncertain effect: number of medicines prescribed  3/4 studies report a reduction in the number of medicines prescribed  1/4 studies reported no change |
|  |  |  |  |  |  |  |  |  | Uncertain effect: Healthcare utilisation  3/5 studies report a reduction in healthcare utilisation  2/5 studies report no change |
|  |  |  |  |  |  |  |  |  | Uncertain effect: Prescribing  3/4 studies reported an improvement in prescribing  1/4 studies reported no change |
|  |  |  |  |  |  |  |  | Patient-orientated | Evidence for effect: adherence  2/2 studies report improved adherence |
|  |  |  |  |  |  |  |  |  | Uncertain effect: Quality of life  3/4 studies report no evidence of an improvement in QoL  1/4 studies report an improvement in QoL |
| Systematic reviews up to and including 2015 | | | | | | | | | |
| Bayoumi 2009 [28] | What is the efficacy of interventions to improve medication reconciliation among community-dwelling adults in primary care settings? | RCT n=1  before and after study n=3 | | Community- dwelling adults;  primary care, ambulatory settings, or in transition into or out of hospital | USA, Canada, Northern Ireland | No | Low | Number of discrepancies in name, dose, and frequency between recorded and patient-orientated medications. | Uncertain effect  3/4 studies reported an increase in the number of medication discrepancies identified  1/4 studies reported no significant change |
|  |  |  |  |  |  |  |  | Clinical relevance of the medication discrepancies detected | Uncertain effect: clinical significance of discrepancies |
| Castelino 2009 [12] | Evaluation of interventions involving pharmacists, directed toward reducing sub- optimal prescribing | RCT n=12 | | Patients ≥ 65;  inpatients, outpatients, and primary care | USA, Canada, Belgium, Australia | No | Critically low | Suboptimal prescribing (overuse, misuse, underuse) | Uncertain effect: inappropriate prescribing  9/11 studies report improved prescribing  2/11 studies report no significant difference |
|  |  |  |  |  |  |  |  | Economic outcomes (hospital services use and healthcare use) | Uncertain effect  1/5 studies reported less hospital use  4/5 studies result not reported |
|  |  |  |  |  |  |  |  | Clinical outcomes (mobility, confusion, pain,  falls, resident  behaviour, adverse drug events) | Uncertain effect  1/5 studies reported better pain control  4/5 studies result not reported |
| Costello 2009 [25] | What is the evidence around medicines management in children | RCT n=2,  Audit n=2, Retrospective comparative study n=1,  Longitudinal Prospective study n=1,  Systematic Review n=1,  Descriptive study n=1 | | Paediatric and adult patients;  community pharmacy, community practice, GP practice, wards/hospital clinics. | UK, NZ, Malta, USA, Japan | No | Critically Low | Clinical outcomes | Uncertain effect: morbidity, hospital readmissions, GP visits, school absence |
|  |  |  |  |  |  |  |  |  | Uncertain effect: Medication-Related Problems |
|  |  |  |  |  |  |  |  | Patient-orientated (e.g., patient knowledge, satisfaction, adherence) | Uncertain effect: patient reported outcomes |
| George 2008 [27] | What is the effectiveness of interventions to improve medication adherence in elderly community dwelling patients prescribed multiple long-term medications? | RCT n=7,  non-randomised trial n=1,  multiphase prospective study n=1 | | Elderly patients;  all settings | Europe, Canada, USA, Australia | No | Critically low | Adherence | Evidence of effect:  4/8 studies reported a significant effect on adherence  4/8 studies did not report significant effect on adherence  Mean relative change in adherence across 8 studies = + 11.4% |
| Geurts 2012 [29] | What is the impact of collaboration between pharmacists and GPs and what are the outcomes on patients’ health? | RCT n=26,  Other studies n=51 | | Variable adult population;  Family practice, general practice, community pharmacy | Europe, USA, Canada, Australia, New Zealand | No | Critically low | Non-specific outcomes on patients’ health | Uncertain effect: hospital admissions  5/9 studies reported decrease in hospital admissions  1/9 studies reported statistically significant increase in hospital admissions  3/9 studies reported no effect |
|  |  |  |  |  |  |  |  |  | Evidence of effect: number of medication-related problems  2/2 studies reported positive effects on MRPs resolved |
|  |  |  |  |  |  |  |  |  | Uncertain effect: improving prescribing of medication  2/6 studies reported a reduction in the number of prescribed medicines  4/6 reported no difference in the number of medicines prescribed  5/5 studies reported an increase in the number of drug changes |
|  |  |  |  |  |  |  |  |  | Uncertain effect: quality-of-life scores  7/10 studies reported evidence of no effect on QoL  3/10 studies report improved QoL |
|  |  |  |  |  |  |  |  |  | Uncertain effect: increasing compliance and patient knowledge |
|  |  |  |  |  |  |  |  |  | Uncertain effect: improving clinical values, e.g., cholesterol levels |
| Hatah 2014 [30] | What is impact of fee-for-service pharmacist-led medication review on patient outcomes and quantify this according to the type of review undertaken? | RCT n=8,  cohort studies n=17,  Prospective before/ after study n=1 | | Elderly or patients with specific diseases;  pharmacy, patients’ home, community health centre, GP clinics | US, UK, Denmark, Germany, Canada, Netherlands, Australia, Chile, Belgium | Yes | Critically low | Mortality | No effect  MA of 5 studies, 771 partipants  **(OR 1.50, 95% CI 0.65, 3.46, P = 0.34)** |
|  |  |  |  |  |  |  |  | Hospitalisation | No effect  MA of 9 studies, 1324 partipants  **(OR 0.69, 95% CI 0.39, 1.21, P = 0.19)** |
|  |  |  |  |  |  |  |  | Clinical biomarkers | Evidence of effect: Improvement in the attainment of target biomarkers for blood pressure  MA of 6 studies, 236 partipants  **(OR 3.50,95% CI 1.58, 7.75, P = 0.002)**  Evidence of effect: Improvement in the attainment of target biomarkers for LDL  MA of 4 studies, 334 participants  **(OR 2.35,95% CI 1.17, 4.72, P = 0.02)** |
|  |  |  |  |  |  |  |  | Medication adherence | Uncertain effect  11/19 studies report improvements in adherence  6/19 studies report no difference  2/19 studies report differing results depending upon how outcome was measured |
|  |  |  |  |  |  |  |  | Economic | Uncertain effect  1/6 studies report reduction in medication costs  2/6 studies favour usual care  3/6 studies report no difference |
|  |  |  |  |  |  |  |  | Quality of life | Uncertain effect  6/13 studies reported improvement in QoL  5/13 studies reported no difference in QoL  2/13 studies reported QoL was better in intervention group |
| Holland 2008 [11] | What are the effects of medication review by pharmacists on substantive clinical outcomes (namely, hospital admissions and mortality) for older people across all care settings? | RCTs n=32 | | Elderly (average across trials 71 years);  Hospital, clinic/primary care setting, community pharmacy, patient’s home, nursing home | UK, USA, Canada, Singapore, Australia | Yes | Critically low | Hospital emergency admission | No effect: all- cause admission  MA of 17 studies, 9,990 participants  **(RR) of 0.99 [95% CI 0.87, 1.14, P <0.91].** |
|  |  |  |  |  |  |  |  | All-cause mortality | No effect  MA of 22 studies, 11,741 participants  **(RR = 0.96, 95% CI 0.82, 1.13, P < 0.65)** |
|  |  |  |  |  |  |  |  | Mean drugs prescribed | Evidence of effect: reduction in number of drugs prescribed  MA of 15 studies, 6,358 partipants  (**Weighted mean difference =-0.48, 95% CI -0.89, -0.07**) Marked heterogeneity (P < 0.001, I^2^= 85.9%) |
|  |  |  |  |  |  |  |  | Cost analysis | Uncertain effect  4/14 studies report significant positive effect  6/14 studies report nonsignificant positive effect  2/14 studies report evidence of no effect  2/14 report evidence of a negative effect |
|  |  |  |  |  |  |  |  | Medication-related problems (MRPs) | Evidence of effect  4/4 studies reported a significant positive effect on number of MRPs |
|  |  |  |  |  |  |  |  | Knowledge | Uncertain effect  8/11 studies report improved knowledge  3/11 studies report no difference |
|  |  |  |  |  |  |  |  | Patient satisfaction | Uncertain effect: satisfaction  2/4 studies report significant positive effect  1/4 studies report nonsignificant positive effect  1/4 studies report negative effect |
|  |  |  |  |  |  |  |  | Quality of Life | Uncertain effect: QoL  4/12 studies report nonsignificant positive effect  8/12 report evidence of no effect |
|  |  |  |  |  |  |  |  | Adherence | Uncertain effect: adherence  11/14 studies report improvement in adherence  3/14 studies report no effect on adherence |
| Kucukarslan 2011 [13] | What is the evidence of the impact of Medication Therapy Management (MTM) services on patient outcomes in order to assist healthcare professionals in improving services for their patients? | RCT n=8 | | Patients >18 years; Clinics, community pharmacy | USA, Canada, UK | No | Critically low | Patient outcomes | Uncertain effect: improvements to Clinical outcomes (e.g., BP, HbA1c LDL) |
|  |  |  |  |  |  |  |  |  | Uncertain effect: Blood Pressure  3/ 4 studies reported a decrease  1/ 4 study reported no effect |
|  |  |  |  |  |  |  |  |  | No effect: adherence  2/2 studies |
|  |  |  |  |  |  |  |  |  | Uncertain effect: symptoms  1/ 2 study reported an improvement in symptoms  1/ 2 study reported no change |
|  |  |  |  |  |  |  |  |  | No effect: Quality of Life  4/4 studies |
|  |  |  |  |  |  |  |  |  | No effect: LDL  2/2 studies |
|  |  |  |  |  |  |  |  |  | Uncertain effect: HbA1c  3/ 4 studies reported an improvement  1/ 4 study report no effect |
|  |  |  |  |  |  |  |  |  | Uncertain effect: patient satisfaction  1/ 2 study reported an increase  1/ 2 reported no effect |
|  |  |  |  |  |  |  |  | Drug or healthcare costs | No effect: healthcare utilisation  1/1 study |
|  |  |  |  |  |  |  |  |  | Uncertain effect: costs  2/4 studies reported an improvement  1/ 4 study reported an increase  1/ 4 study reported no effect |
| Kwint [14] | How does the extent of collaboration between the general practitioner (GP) and the pharmacist impact on the implementation of recommendations arising from medication review? | RCT n=12 | | Older adults, mean age 70 years;  home-dwelling, primary care | Canada, New Zealand, Netherlands, USA, UK, Australia | Yes +  +Not for outcomes | Critically low | Implementation rate of recommendations following identification of medicines-related problems (MRPs) | Mean implementation rate of recommendations was 50 % (range 17–86) (12 studies). |
|  |  |  |  |  |  |  |  | Clinical outcomes | No effect: quality of life  5/6 studies reported no effect on QoL  1/6 studies reported negative effects in some domains  No effect on hospital admissions  4/4 studies reported evidence of no effect |
|  |  |  |  |  |  |  |  | Intermediate outcomes (e.g., adherence) | No effect: adherence  2/2 studies reported evidence of no effect |
|  |  |  |  |  |  |  |  | Process outcomes (e.g., drug changes, number of drugs) | Uncertain evidence: Process outcomes  2/6 studies reported a reduction in number of prescribed medicines  4/6 studies report no evidence of effect  5 studies reported an increase in number of drug changes |
| Rollason 2003 [24] | What is the role of pharmacists and pharmacist interventions in reducing polypharmacy | RCT n=7,  controlled study n=7 | | Elderly patients (mean age across studies 64 - 86);  outpatients, nursing home, inpatient, post discharge | USA, Canada, Belgium, UK, Korea | No | Critically low | Reduction in number of medications | Evidence of effect: reduction in number of medicines  7/14 studies reported a significant reduction in number of medicines  1/14 studies reported no difference between control and intervention  6/14 studies not reported |
| Royal 2006 [26] | What interventions delivered in primary care settings reduce preventable drug related morbidity? | RCT n= 29,  Controlled before/after study n=8,  retrospective study n=1 | | All patient populations; general practice | USA, Europe, Australia, New Zealand | Yes | Critically low | Reduce drug-related morbidity | No significant effect: falls  MA of 9 studies, 4,748 participants  **(OR 0.91 (95% CI 0.68 to 1.21)** |
|  |  |  |  |  |  |  |  | Hospitalisation | Evidence of effect: reduction on hospital admission  MA of 13 studies, 20,318 participants  (OR 0.64 (95% CI 0.43 to 0.96) but significant heterogeneity; sensitivity analysis reduces the size of effect **OR 0.92, 95% CI 0.81 to 1.05** |
| Tan 2014 [15] | What is the effectiveness of clinical pharmacist services delivered in primary care general practice clinics | RCT n=38 | | Adults;  General practice | USA, UK, Canada, South America, Asia | Yes | Critically Low | Appropriateness of prescribing | Evidence of effect: improving quality of prescribing and medication appropriateness |
|  |  |  |  |  |  |  |  | Medication use | Evidence of effect: positive effect on resolution of medication-related problems |
|  |  |  |  |  |  |  |  | Health service use | No effect |
|  |  |  |  |  |  |  |  | Clinical, functional, practice or economic outcomes | Evidence of effect: Systolic BP reduction  MA of 11 studies, No. of participants not reported  **-5.72 mm Hg (95% CI, -7.05 to -4.39 P<0.001** |
|  |  |  |  |  |  |  |  |  | Evidence of effect : Diastolic BP reduction  MA of 11 studies, No. of participants not reported  **-3.47 mm Hg, (95% CI -4.35 to -2.58, P<0.001)** |
|  |  |  |  |  |  |  |  |  | Evidence of effect : HbA1C reduction  MA of 5 studies, No. of participants not reported  **-0.88% (95%CI, -1.15 to -0.62, P<0.001)** |
|  |  |  |  |  |  |  |  |  | Evidence of effect: LDL-cholesterol reduction  MA of 3 studies, No. of participants not reported  **-18.72 mg/dL (95% CI, -34.10 to -3.36, P <0.017)** |
|  |  |  |  |  |  |  |  |  | No effect: quality of life or patient satisfaction. |
| Viswanathan 2015 [31] | What is the effect of a Medication Therapy Management (MTM) service in outpatient settings | RCT n=21,  Non-RCT n=4,  Cohort study n=19 | | All patient populations; ambulatory settings | USA, Australia, UK, Canada, Brazil | Yes | Critically low | Health outcomes | Uncertain evidence: clinical outcomes e.g., HbA1c, lipids, BP, anticoagulation. |
|  |  |  |  |  |  |  |  | Mortality | Uncertain evidence: mortality  3/3 studies report insufficient evidence  OR for studies 0.5-0.92 with wide confidence intervals |
|  |  |  |  |  |  |  |  | Patient-centred functioning | Uncertain evidence: cognitive and affective function  3/3 studies report insufficient evidence |
|  |  |  |  |  |  |  |  | Quality of life | No effect health-related Quality of Life  3/3 studies report evidence of no effect |
|  |  |  |  |  |  |  |  | Satisfaction | No effect  3/3 studies report evidence of no effect |
|  |  |  |  |  |  |  |  | Health care use and costs. | Uncertain effect: hospitalisations/ outpatient visits.  MA of 3 studies, 2,208 participants  standardized mean difference of outpatient appointments, **0.05; 95%CI, −0.03 to 0.13; P = 0.25**  Weighted mean difference for hospitalisations **0.04; 95%CI, −0.01 to 0.08; P =0 .09**  Uncertain effect: medication costs. |
| **Key:** | No effect | |  | Evidence of no effect was reported as quantitative data. In the absence of a meta-analysis this information was extracted from authors’ conclusions. | | | | LDL= low density lipoprotein  CVD= cardiovascular disease  BP = blood pressure  CMR= Clinical Medication Review  MRPs= Medication-related problems; also known as drug-related problems  MD= Mean difference  MA= Meta-analysis  RCT= Randomised Controlled Trial  QoL= Quality of Life | |
|  | Uncertain effect | |  | This was reported when the systematic review included studies with positive and negative effects in outcomes, leaving uncertainty about the effect of medication reviews on a particular outcome measure | | | |  |  |
|  | Evidence of effect | |  | Evidence of effect could be positive, in favour of the medication review, or negative, in favour of the control group. Evidence of effect was reported as quantitative data. In the absence of a meta-analysis this information was extracted from authors’ conclusions | | | |  |  |
