# Supplementary material for: Pharmacist-led medication reviews: A scoping review of systematic reviews
Source: PLoS One. 2024 Sep 6;19(9):e0309729. doi: 10.1371/journal.pone.0309729 (PMC11379247; doi:10.1371/journal.pone.0309729)
Supplement: S2 Table — (DOCX) [file pone.0309729.s003.docx]

| **S3 Table 2 Complete AMSTAR 2 assessment** |
| --- |
| \| Author (year) \| 1.Did the research questions and inclusion criteria for the review include the components of PICO? \| *2.Did the report of the review contain an explicit statement that the review methods were established prior to the conduct of the review and did the report justify any significant deviations from the protocol?* \| 3.Did the review authors explain their selection of the study designs for inclusion in the review \| *4.Did the review authors use a comprehensive literature search strategy?* \| 5.Did the review authors perform study selection in duplicate? \| 6.Did the review authors perform data extraction in duplicate? \| *7.Did the review authors provide a list of excluded studies and justify the exclusions?* \| 8.Did the review authors describe the included studies in adequate detail? \| *9.Did the review authors use a satisfactory technique for assessing the risk of bias (RoB) in individual studies that were included in the review?* \| 10.Did the review authors report on the sources of funding for the studies included in the review? \| *11.If meta-analysis was performed did the review authors use appropriate methods for statistical combination of results?* \| 12.If meta-analysis was performed, did the review authors assess the potential impact of RoB in individual studies on the results of the meta-analysis or other evidence synthesis? \| *13.Did the review authors account for RoB in individual studies when interpreting/ discussing the results of the review?* \| 14.Did the review authors provide a satisfactory explanation for, and discussion of, any heterogeneity observed in the results of the review? \| *15.If they performed quantitative synthesis did the review authors carry out an adequate investigation of publication bias (small study bias) and discuss its likely impact on the results of the review?* \| 16.Did the review authors report any potential sources of conflict of interest, including any funding they received for conducting the review? \| **OVERALL RATING** \| \| --- \| --- \| --- \| --- \| --- \| --- \| --- \| --- \| --- \| --- \| --- \| --- \| --- \| --- \| --- \| --- \| --- \| --- \| \| Post 2015 systematic reviews \| \| \| \| \| \| \| \| \| \| \| \| \| \| \| \| \| \| \| Alldred 2016 [16] \| Yes \| Yes \| No \| Yes \| Yes \| Yes \| Yes \| Yes \| Yes \| Yes \| No MA \| No MA \| Yes \| Yes \| No MA \| Yes \| High \| \| Bulow 2023 [21] \| Yes \| Yes \| Yes \| Yes \| Yes \| Yes \| Yes \| Yes \| Yes \| Yes \| No MA \| No MA \| Yes \| Yes \| No MA \| Yes \| High \| \| Al- babtain 2022 [20] \| Yes \| Yes \| No \| Yes \| Yes \| Yes \| Yes \| No \| Yes \| Yes \| Yes \| No \| No \| Yes \| Yes \| Yes \| Moderate \| \| Atey 2022 [34] \| Yes \| Yes \| No \| Partial Yes \| Yes \| Yes \| Partial Yes \| Yes \| Yes \| No \| Yes \| Yes \| Yes \| Yes \| Yes \| Yes \| Moderate \| \| Fadaleh 2022 [22] \| Yes \| Yes \| Yes \| Partial Yes \| Yes \| Yes \| Partial Yes \| Partial Yes \| Partial Yes \| No \| Yes \| No \| Yes \| Yes \| Yes \| Yes \| Moderate \| \| Martinez-Mardones 2019 [19] \| Yes \| No \| No \| Yes \| Yes \| Yes \| Partial Yes \| Partial Yes \| Partial Yes \| No \| Yes \| Yes \| Yes \| Yes \| Yes \| Yes \| Low \| \| Ahumada-Canale 2019 [18] \| No \| Yes \| No \| Partial Yes \| Yes \| No \| Partial Yes \| Yes \| Partial Yes \| Yes \| No MA \| No MA \| No \| No \| No MA \| Yes \| Critically Low \| \| Bou Malham 2021 [33] \| Yes \| No \| No \| Partial Yes \| Yes \| Yes \| Partial Yes \| Yes \| No \| Yes \| Mo MA \| No MA \| No \| No \| No MA \| Yes \| Critically Low \| \| Hikaka 2019 [23] \| Yes \| No \| No \| Yes \| Yes \| No \| No \| Partial Yes \| No \| No \| Mo MA \| No MA \| No \| No \| No MA \| Yes \| Critically Low \| \| Huiskes 2017 [17] \| Yes \| No \| No \| Partial Yes \| Yes \| Yes \| No \| Partial Yes \| Partial Yes \| No \| Yes \| Yes \| Yes \| Yes \| No \| Yes \| Critically Low \| \| Jokanovic 2016 [32] \| No \| No \| No \| Yes \| Yes \| Yes \| No \| Yes \| Partial Yes \| Yes \| No MA \| No MA \| Yes \| No \| No MA \| Yes \| Critically Low \| \| Before 2015 systematic reviews \| \| \| \| \| \| \| \| \| \| \| \| \| \| \| \| \| \| \| Bayoumi 2009 [28] \| Yes \| No \| No \| Partial Yes \| Yes \| Yes \| Yes \| Partial Yes \| Yes \| No \| No MA \| No MA \| Yes \| Yes \| No MA \| Yes \| Low \| \| Castelino 2009 [12] \| No \| No \| No \| Partial Yes \| No \| No \| No \| No \| No \| No \| No MA \| No MA \| No \| No \| No MA \| No \| Critically Low \| \| Costello 2009 [25] \| No \| No \| No \| Yes \| No \| No \| No \| Partial Yes \| No \| No \| No MA \| No MA \| No \| No \| No MA \| Yes \| Critically Low \| \| George 2008 [27] \| Yes \| No \| No \| Partial Yes \| Yes \| Yes \| Yes \| Partial Yes \| No \| No \| No MA \| No MA \| No \| No \| No MA \| Yes \| Critically Low \| \| Geurts 2012 [29] \| No \| No \| No \| Partial Yes \| Yes \| Yes \| No \| No \| No \| No \| No MA \| No MA \| No \| No \| No MA \| Yes \| Critically Low \| \| Hatah 2014 [30] \| Yes \| No \| Yes \| Partial Yes \| Yes \| Yes \| No \| Partial Yes \| Partial Yes \| No \| Yes \| Yes \| Yes \| Yes \| Yes \| Yes \| Critically Low \| \| Holland 2008 [11] \| Yes \| No \| Yes \| Partial Yes \| Yes \| Yes \| No \| Yes \| Partial Yes \| No \| Yes \| Yes \| Yes \| Yes \| Yes \| Yes \| Critically Low \| \| Kucukarslan 2011 [13] \| No \| No \| No \| Partial Yes \| No \| No \| No \| No \| No \| No \| No MA \| No MA \| Yes \| No \| No MA \| Yes \| Critically Low \| \| Kwint [14] \| No \| No \| No \| Partial Yes \| Yes \| Yes \| Yes \| No \| Partial Yes \| No \| Yes \| Yes \| Yes \| No \| No \| Yes \| Critically Low \| \| Rollason 2003 [24] \| No \| No \| No \| No \| No \| No \| No \| Partial Yes \| No \| No \| No MA \| No MA \| No \| No \| No MA \| Yes \| Critically Low \| \| Royal 2006 [26] \| No \| No \| Yes \| Yes \| Yes \| Yes \| No \| Partial Yes \| Partial Yes \| No \| Yes \| Yes \| Yes \| Yes \| Yes \| Yes \| Critically Low \| \| Tan 2014 [15] \| No \| No \| No \| Yes \| Yes \| Yes \| No \| Partial Yes \| Yes \| No \| Yes \| Yes \| Yes \| Yes \| No \| No \| Critically Low \| \| Viswanathan 2015 [31] \| Yes \| Yes \| Yes \| Partial Yes \| Yes \| Yes \| No \| No \| Partial Yes \| No \| Yes \| Yes \| Yes \| Yes \| No \| Yes \| Critically Low \| |
